# On the Estimation of Heritability with Family-Based and Population-Based Samples

**DOI:** 10.1155/2015/671349

**Published:** 2015-08-03

**Authors:** Youngdoe Kim, Young Lee, Sungyoung Lee, Nam Hee Kim, Jeongmin Lim, Young Jin Kim, Ji Hee Oh, Haesook Min, Meehee Lee, Hyeon-Jeong Seo, So-Hyun Lee, Joohon Sung, Nam H. Cho, Bong-Jo Kim, Bok-Ghee Han, Robert C. Elston, Sungho Won, Juyoung Lee

**Affiliations:** ^1^The Center for Genome Science, Korea National Institute of Health, KCDC, Osong 361-951, Republic of Korea; ^2^Department of Applied Statistics, Chung-Ang University, Seoul 156-756, Republic of Korea; ^3^Interdisciplinary Program in Bioinformatics, Seoul National University, Seoul 151-742, Republic of Korea; ^4^Chunlab Inc., Seoul National University, Seoul 151-742, Republic of Korea; ^5^Department of Epidemiology, Seoul National University, Seoul 151-742, Republic of Korea; ^6^Department of Preventive Medicine, Ajou University School of Medicine, Suwon 443-380, Republic of Korea; ^7^Department of Epidemiology and Biostatistics, Case Western Reserve University, Cleveland, OH 44106-7281, USA; ^8^Department of Epidemiology and Biostatistics, School of Public Health & Institute of Health and Environment, Seoul National University, Seoul 151-742, Republic of Korea

## Abstract

For a family-based sample, the phenotypic variance-covariance matrix can be parameterized to include the variance of a polygenic effect that has then been estimated using a variance component analysis. However, with the advent of large-scale genomic data, the genetic relationship matrix (GRM) can be estimated and can be utilized to parameterize the variance of a polygenic effect for population-based samples. Therefore narrow sense heritability, which is both population and trait specific, can be estimated with both population- and family-based samples. In this study we estimate heritability from both family-based and population-based samples, collected in Korea, and the heritability estimates from the pooled samples were, for height, 0.60; body mass index (BMI), 0.32; log-transformed triglycerides (log TG), 0.24; total cholesterol (TCHL), 0.30; high-density lipoprotein (HDL), 0.38; low-density lipoprotein (LDL), 0.29; systolic blood pressure (SBP), 0.23; and diastolic blood pressure (DBP), 0.24. Furthermore, we found differences in how heritability is estimated—in particular the amount of variance attributable to common environment in twins can be substantial—which indicates heritability estimates should be interpreted with caution.

## 1. Introduction

Under polygenic inheritance, the effects of segregation at single loci are assumed to be too small to estimate individually and the total genetic variance has been considered to identify the overall genetic effect underlying a trait. Genetic variance consists of additive, dominant, and epistatic components. However, the amount of dominant variance is usually assumed to be relatively small compared to the additive variance and is never identified without a family-based sample that includes bilineal relatives. Similarly, estimation of the epistatic variance (which may include additive components) requires special relationships in family data and is also assumed to be small. Therefore, the estimation of genetic variance has been confined to the additive genetic variance and, to estimate heritability, the proportion of the phenotypic variance attributable to only additive genetic variance has been used even though this can lead to biased estimation in the presence of dominant variance, epistatic variance, and gene × environmental interaction [1].

In general, a parameter allowing for additive polygenic variance can be incorporated into the phenotypic covariances between pairs of individuals, and there are two main ways for incorporating this parameterization. In the absence of population substructure, dominance or any environmental effect shared by family members, the phenotypic covariances can be expressed as a function of the kinship coefficient between family members in family-based samples. Under this parameterization, the additive polygenic variance is obtained from the covariances between family members using variance component models [2–5]. Alternatively, since the advent of large-scale genome data, which reveals similarity in genotypic background, the genetic relationships between individuals have become estimable from genome-wide data and this has also been used to identify population substructure. In the same context, the phenotypic variance explained by additive polygenic variance can also be estimated in population-based samples from the genetic relationships obtained in this way [6, 7]. In particular, the individuals in population-based samples are not closely related and share much less common environmental exposures than do the family members in family-based samples. For this reason Yang et al. [8] suggested excluding closely related individuals from the analysis when estimating heritability from population-based samples, noting that the environmental effect shared by family members seems to be inversely related to their degree of physical proximity, so that close relatives inflate any estimate of heritability.

In this paper, motivated by wishing to calculate the heritability of cardiovascular disease related traits in the Korean population, we examine to what extent estimates of heritability depend on how they are estimated. We calculate the heritability of various traits related to cardiovascular disease in a Korean population using two family-based cohorts, the healthy Twin Study, Korea (HTK) [9] and Ansung Family (ASF) cohorts, and one population-based cohort, that for the Korean Association Resource (KARE) [10] project. Comparing the heritability estimates from family-based and population-based samples, disturbing differences were found. With simulation studies we show that the meaning of heritability estimates can be affected by the absence of highly correlated samples and be substantially inflated by variance attributable to common environment. Thus heritability estimates should be interpreted with caution.

## 2. Materials

Three cohorts, all part of the Korean Genome Epidemiology Study (KoGES) which is an ongoing prospective epidemiological study, have been utilized to estimate heritability: the KARE project [10] cohort and the HTK [9] and ASF cohorts. These cohorts were genotyped in the Korean Genome Analysis Project (KoGAP) by the Center for Genome Science in the Korea Center for Disease Control and Prevention, which was launched in Korea between 2001 and 2007.

### 2.1. KARE Project

The KARE project, with 10,038 participants who were living in Ansung (rural) and Ansan (urban), was initiated in 2007 for large-scale genome-wide association studies (GWAS) based on the Korean population. Among the 10,038 participants, 10,004 individuals were genotyped for 500,568 SNPs with the Affymetrix Genome-Wide Human SNP array 5.0. We discarded SNPs with *P* values for departure from Hardy-Weinberg equilibrium (HWE) less than 10^−5^, with genotype call rates less than 95%, or minor allele frequencies (MAF) less than 0.01, leaving 350,364 SNPs for subsequent analysis. Individuals with low call rates (<95%, *n* = 401), high heterozygosity (>30%, *n* = 11), gender inconsistencies (*n* = 41), or serious concomitant illness (*n* = 101) were excluded from analysis, along with 601 individuals related or identical whose computed average pairwise identical in state value was higher than that estimated from first-degree relatives of Korean sib-pair samples (>0.8). In total 8,842 individuals were analyzed. In 20 randomly selected duplicate samples, we found that genotype concordance rates exceeded 99.7%, with no single SNP excessively discordant.

### 2.2. HTK Cohort

The HTK cohort was initiated to identify genetic variation responsible for complex traits as well as the role of the environment in the etiology of complex diseases. Some healthy twins in this cohort were recruited through advertisements in a nationwide newspaper and through posters in about 300 hospitals. Other twin families were selected from the large Korean Genomic Cohort Study of adult individuals and the KoGAP. Then the family members of the selected twins were recruited into this cohort. It should be noted that health status was not considered for sampling. This type of family study can be useful for detecting quantitative trait loci and genetic variations underlying common diseases [11]. Among the 2,473 participants enrolled from April 2005 to December 2008, there are 990 individuals comprising monozygotic (MZ) twins and 234 individuals comprising dizygotic (DZ) twins, and 1861 of these individuals could be genotyped with the Affymetrix Genome-Wide Human SNP array 6.0. We discarded SNPs with *P* values for departure from HWE less than 10^−5^ or MAF less than 0.01. In addition, SNPs were excluded if Mendelian errors or double recombinants were found in at least 3 families, and in total 520,484 SNPs were used for analysis. We calculated the proportion of genotypes identical in state between individuals in each family and excluded those with any inconsistency between the genetic and reported relationship (*n* = 58). Also, individuals who had coding errors for MZ/DZ status (*n* = 2) were excluded, and as a result genotypes for 1801 family members were available for analysis. Among the genotyped individuals, there are 4 pairs of MZ twins and 393 genotyped individuals whose MZ twin siblings were not genotyped. Also 84 pairs of DZ twins were genotyped, and there are 16 additional genotyped individuals whose DZ twin siblings' genotypes were unknown. There are 162 nuclear families and 3 families consisting of individuals in three generations that include MZ/DZ twins.

### 2.3. ASF Cohort

In the Ansung area, 5,018 unrelated and related participants were initially recruited for the KARE project; another cohort to study type 2 diabetes was initiated in this area in 2007. In this cohort, some individuals were selected from the KARE project, and their family members and other individuals from the Ansung area who were not in the KARE project were included, if they were diagnosed as having type 2 diabetes and agreed to participate in this study. This sampling scheme could lead to the presence of ascertainment bias, but the small correlations between type 2 diabetes status and the traits of interest (see Table S1 in Supplementary Material available online at http://dx.doi.org/10.1155/2015/671349) reveal that any ascertainment bias would not be substantial. In these samples, 456 individuals who were included in the KARE project were genotyped with the Affymetrix Genome-wide Human SNP array 5.0, and another 781 individuals were genotyped with the Affymetrix Genome-wide Human SNP array 6.0. Individuals were excluded if they reported relationships in the family inconsistent with the genotypic relationships estimated by the proportion of genotypes identical in state (*n* = 41) or had unavailable trait data (*n* = 412). Also, SNPs were excluded if Mendelian inconsistency was found in at least 3 families, the *P* values for HWE were less than 10^−5^, or the MAF was less than 0.01. As a result, 784 family members with 417,719 SNPs were used for our analysis.

## 3. Methods

To estimate heritability we used the freely available software Genome-wide Complex Trait Analysis (GCTA) [8] and the ASSOC program in the Statistical Analysis for Genetic Epidemiology (S.A.G.E.) [12] package. We considered eight traits: height, body mass index (BMI), triglycerides (TG), total cholesterol (TCHL), high-density lipoprotein (HDL), low-density lipoprotein (LDL), systolic blood pressure (SBP), and diastolic blood pressure (DBP). We included age, age^2^, and sex as covariates. In particular, the linear mixed model for GCTA is robust to population substructure, and the EIGENSTRAT method [13] which includes PC scores as covariates was not applied. The effect of a living environment variable (urban versus rural) was not significant at the 0.05 significance level for any of the eight traits and so was not included as a covariate in the detailed analyses reported in Tables S2–S9. Quantile-quantile plots in Figures S1-S2 indicate that TG is not normally distributed and log-transformed TG (log TG) was used to obtain approximate normality. For the other phenotypes, the original scales were used because heritability estimates on the original scale and after inverse-normal transformation were almost the same and interpretation is not straightforward for the inverse-normal transformed data. The missing rates for each phenotype were calculated (see Table S10) and were usually very small. ASSOC parameterizes the phenotypic correlations between individuals using the reported familial relationships and can split the nonpolygenic variance into components for measurement error, sibling, and marital effects, and these results were summarized. GCTA estimates heritability by parameterizing phenotypic correlations with the estimated genetic relationship matrix (GRM) from the standardized genotypes. In particular, the results from GCTA were obtained with and without the default GRM-cutoff option. In addition, we separately analyzed monozygotic (MZ) and dizygotic (DZ) twin data, to estimate the relative proportion of the phenotypic variance attributable to common environmental effects.

### 3.1. Heritability Estimation Using Familial Relationships

Under the multivariate normality model, the covariance between family members can be expressed as a function of their kinship coefficients and this can be utilized to estimate heritability. We estimated the heritability from the family data, separately in the HTK and ASF cohorts, with the ASSOC program in S.A.G.E. (ver. 6.2) [12]. ASSOC is based on a linear mixed model and the parameters are estimated by the maximum likelihood (ML) method. Let *y*_*ij*_ denote the response for individual *j* in family *i*, where *i* = 1,…, *n* and *j* = 1,…, *n*_*i*_; *n* and *n*_*i*_ indicate the number of families and the number of individuals in family *i*, respectively. Also, let *x*_*ij*_ indicate covariates that affect *y*_*ij*_. Then, denoting *π*_*ijj*′_ as the kinship coefficient between individual *j* and individual *j*′ in family *i*, we let

(1) Xixi1⋮xini,Yi=yi1⋮yini,Φi=12πi12⋯2πi211⋱⋮⋱⋱.

We denote the additive polygenic, dominant polygenic, and random error variances, respectively, by *σ*_*a*_^2^, *σ*_*d*_^2^, and *σ* ^2^. If we also denote the *w* × *w* identity matrix by **I**_*w*_, the linear model used in ASSOC for random mating and only additive effects is

(2) $$\begin{array}{c} Y_{i}=X_{i}\beta +\varepsilon_{i},\;\text{where}\;\;\varepsilon_{i}\sim \text{MVN}\left(0,\sigma^{2}I_{n_{i}}+\sigma_{a}^{2}\Phi_{i}\right). \end{array}$$

**Ф**_*i*_ will be called the familial relationship matrix (FRM) in the remainder of this paper. Furthermore, ASSOC can estimate the variances separately attributable to polygenic, common sibship, and marital effects as described by Elston et al. [14].

S.A.G.E. ASSOC was used to estimate heritability in the family-based HTK and ASF cohorts and, for a fair comparison with GCTA, only genotyped individuals were analyzed this way. In the HTK cohort, 1801 genotyped individuals were considered, and there are 4 pairs of MZ twins among those genotyped. S.A.G.E. cannot easily handle MZ twins, and a single individual for each MZ twin was randomly selected for analysis with both ASSOC and GCTA. There is other software available that can handle MZ twins [15–17] in pedigrees, but this was not considered because the number of genotyped MZ twins is very small and so the variance attributable to common environment could not be well estimated in these cohorts using the GRM. We used the program PEDINFO in S.A.G.E. to provide descriptive statistics of the pedigree data.

### 3.2. Heritability Estimation Using Estimated Genetic Relationships

When large-scale genotypes are available, the GRM can be estimated with the software GCTA [8] and, instead of the FRM, the estimated GRM can be incorporated into the same linear mixed model (2) as available in ASSOC, to estimate *σ*_*a*_^2^. The minor allele frequencies for GRM were estimated by using all individuals even when some individuals were correlated. Because the genetic relationship is estimated with genotypes, GCTA can be applied to both family-based and population-based samples. In addition, the GCTA program can estimate the variance components by both the restricted maximum likelihood (REML) and ML methods. The REML method provides more unbiased estimates of the variance components than the ML method. Therefore we estimated heritability by the REML method when applying GCTA to the KARE project, the HTK cohort, and the ASF cohort, though for these large samples the difference in the estimates is expected to be trivially small. Yang et al. [8] suggested excluding closely related individuals from the analysis when estimating genetic variation captured by all the SNPs, using a GRM-cutoff option. However, for the analysis of family-based samples, family members are highly correlated and most individuals become excluded from the analysis if the GRM-cutoff option for individual selection is activated. We report the results of both with and without the GRM-cutoff option, and we used 0.025 as the GRM-cutoff.

### 3.3. Estimating Familial Correlations with S.A.G.E

FCOR in S.A.G.E. [12] can estimate familial correlations for all pair types existing in a set of pedigrees. FCOR cannot handle the effect of covariates, and thus for height, BMI, log TG, TCHL, HDL, LDL, SBP, and DBP, we calculated the residuals from the linear model with age, age^2^, and sex as covariates. Residuals from this linear model were used to estimate the empirical correlations between family members and their 95% confidence intervals with FCOR in S.A.G.E.

### 3.4. Estimating Variance Attributable to the Common Environment with Twins

If we assume an additive model with no interaction, the phenotypic variance consists of the genetic variance and a common environmental variance component. However, the variance for environmental effects shared by family members is in general unidentifiable. If we further assume that the amount of covariance between MZ twins attributable to a common environmental effect is similar to that between DZ twins [18] and that any dominant or epistatic polygenic effects are relatively small compared to the additive genetic and common environmental effects, the covariance attributable to the common environmental effect can be estimated.

We separated out all the MZ and DZ twins, whether genotyped or not, from the HTK cohort, so that the members in each family are always either MZ or DZ twins in this analysis. In total, 958 individuals (479 pairs) comprising MZ twins and 224 individuals (112 pairs) comprising DZ twins were analyzed. If we denote the common environmental variance by *σ*_*c*_^2^, the polygenic model provides the following variance-covariance structure between twins:

(3) $$\begin{array}{c} \mathrm{cov}\left(\;y_{i1},y_{i2}\;\right)=\left\{\begin{array}{cc} \sigma_{c}^{2}+\sigma_{a}^{2}+\sigma_{d}^{2} & \text{for MZ twins}\\ \sigma_{c}^{2}+0.5\sigma_{a}^{2}+0.25\sigma_{d}^{2} & \text{for DZ twins}. \end{array}\right. \end{array}$$

To construct this variance-covariance structure for MZ and DZ twins in our linear mixed model, we denote *σ*_*Y*_^2^ = var(*y*_*ij*_), *r*_DZ_ = (*σ*_*c*_^2^ + 0.5*σ*_*a*_^2^ + 0.25*σ*_*d*_^2^)/*σ*_*Y*_^2^, and *r*_MZ_ = (*σ*_*c*_^2^ + *σ*_*a*_^2^ + *σ*_*d*_^2^)/*σ*_*Y*_^2^. We define two matrices **A** and **B** as follows:

(4) Aaij,aij=1i,j  are  MZ  twins0otherwise,B=bij,bij=1DZ  twins0otherwise.

Then, our linear model becomes

(5) Y=Xβ+ε,ε~MVN0,σY2V,  where  V=In+rMZA+rDZB.

Here *r*_MZ_ and *r*_DZ_ should be between −1 and 1. We used the REML method to estimate variance parameters, and each parameter was estimated by the average information method [19, 20]. R code for the proposed method can be downloaded from http://healthstat.snu.ac.kr/data/heritability_Rcode.zip. It is simple to show that, ignoring any epistatic effects, 2*σ*_DZ_ − *σ*_MZ_ is *σ*_*c*_^2^ − 0.5*σ*_*d*_^2^ and, if we assume that *σ*_*d*_^2^ = 0, 2*σ*_DZ_ − *σ*_MZ_ becomes *σ*_*c*_^2^ and the proportion of variance attributable to common environment, *ρ*_*c*_, can be calculated by 2*r*_DZ_ − *r*_MZ_. If we let **P** = **V** ^−1^ − **V** ^−1^ **X**(**X** ^*t*^ **V** ^−1^ **X**)^−1^ **X** ^*t*^ **V** ^−1^, the Fisher information matrix for *σ* ^2^, *r*_MZ_, and *r*_DZ_ can be obtained by

(6) $$\begin{array}{c} \Psi =\left(\begin{array}{ccc} \frac{n-p}{2\sigma^{4}} & \frac{1}{2\sigma^{2}}\mathrm{tr}\left(PA\right) & \frac{1}{2\sigma^{2}}\mathrm{tr}\left(PB\right)\\ \frac{1}{2\sigma^{2}}\mathrm{tr}\left(PA\right) & \frac{1}{2}\mathrm{tr}\left(PAPA\right) & \frac{1}{2}\mathrm{tr}\left(PAPB\right)\\ \frac{1}{2\sigma^{2}}\mathrm{tr}\left(PB\right) & \frac{1}{2}\mathrm{tr}\left(PAPB\right) & \frac{1}{2}\mathrm{tr}\left(PBPB\right) \end{array}\right), \end{array}$$

where *n* is a sample size and *p* is the number of covariates. Thus the variance of *ρ*_*c*_ can be obtained by (0, −1,2)Ψ^−1^(0, −1,2)^*t*^. Provided the environmental correlation is the same for both MZ and DZ twins, this estimate can be utilized as a lower bound for the variance attributable to the environmental effects shared by siblings.

### 3.5. Simulation Studies

With extensive simulation studies, we investigated the accuracy of heritability estimates for various scenarios. We generated 5000 pairs of individuals with 100,000 SNPs, and heritability was estimated by GCTA without activating the GRM-cutoff. The individuals in different pairs were generated to be independent and the correlations of genotypes, *r*, between individuals in each pair were generated to be 1/2,1/4,…, 1/128, or 0. A pair of individuals with *r* = 1/2 indicates siblings or a parent-offspring pair. To generate pairs of individuals with correlation of genotypes *r*, randomly selected alleles from two individuals were generated to be identical by descent with probability *r*/2. The minor allele frequencies were generated from *U*(0,0.4) and genotypes were generated with the binomial distribution under Hardy-Weinberg equilibrium (HWE). Monomorphic variants were excluded from the analyses, and all markers were assumed to be in linkage equilibrium. If there are too many redundant SNPs in linkage disequilibrium with the causal variants, the empirical standard deviation of heritability estimates can be inflated and the analysis with GCTA should be modified as indicated by Speed et al. [21].

The traits were generated by summing a polygenic effect and a random effect. The random effect was generated from *N*(0, *σ* ^2^). To create a polygenic effect we simulated 100 independent causal SNPs and we assumed that all or 50 randomly selected ones of these causal SNPs were genotyped. The additive disease mode of inheritance was assumed and a single SNP genetic effect is denoted by *β*_*l*_. Letting *p*_*l*_ be the allele frequency for causal SNP *l*  (*l* = 1,2,…, 100) and heritability be *h* ^2^, the genetic effect, *β*_*l*_, was calculated as

(7) $$\begin{array}{c} \beta_{l}=\sqrt{\frac{h^{2}\sigma^{2}}{200p_{l}\left(1-p_{l}\right)\left(1-h^{2}\right)}}. \end{array}$$

Here *p*_*l*_ were generated from *U*(0,0.1) or *U*(0.1,0.4), respectively, and the genetic effects 2*β*_*l*_^2^ *p*_*l*_(1 − *p*_*l*_), for the 100 causal SNPs, were taken to be equal. *σ* ^2^ was assumed to be 1 and *h* ^2^ was taken to be 0.1, 0.3, 0.5, 0.7, or 0.9.

## 4. Results

### 4.1. Estimates of Heritability in a Korean Population

Table 1 shows the descriptive statistics for eight traits: height, BMI, log TG, TCHL, HDL, LDL, SBP, and DBP. Interquartile ranges for these traits show that the traits in the three cohorts are comparable. We calculated heritabilities in the HTK, ASF, and KARE cohorts separately, and they were also combined to calculate overall heritabilities by pooling the samples and including two dummy (0/1) covariates to adjust for the effects of each sample.  Table 2 shows that the heritability estimates from the pooled samples with GCTA were, for height, 0.60; BMI, 0.32; log TG, 0.24; TCHL, 0.30; HDL, 0.38; LDL, 0.29; SBP, 0.23; and DBP, 0.24. In each case these heritability estimates are between the limits of those from the individual KARE, HTK, and ASF cohorts. Tables S2–S9 show that, in the samples where both GCTA and S.A.G.E. can be applied, the heritability estimates from S.A.G.E. and GCTA are usually comparable. GCTA estimates heritability with the REML method based on an estimated GRM, while S.A.G.E. estimates heritability with the ML method based on the FRM. The estimates from the REML and ML methods must be very similar for a large sample size, and thus the convergence of the estimated GRM to FRM [22] explains their similarity.

### 4.2. Overestimation of Heritability in Family-Based Samples

From Table 2, we see substantial differences between the heritability estimates from population-based samples and those from family-based samples. Our estimates with population-based samples, KARE, are, for height, 0.32; BMI, 0.15; log TG, 0.21; TCHL, 0.18; HDL, 0.16; LDL, 0.16; SBP, 0.26; and DBP, 0.21 (with the living area variable (urban/rural) included as a covariate; for height, 0.32; BMI, 0.15; log TG, 0.24; TCHL, 0.15; HDL, 0.16; LDL, 0.13; SBP, 0.22; and DBP, 0.16). The largest difference between the family-based and population-based samples was found for HDL, followed by height. However, the phenotypic variances are usually similar and so it is unlikely there exists heterogeneity of heritability between the two types of sample. (It should be noted that the probands in ASF were selected from KARE.) Alternatively, these differences could be explained by the different properties of family-based and population-based samples. The variance attributable to the shared environmental effects by family members was estimated for HTK and ASF with ASSOC. Significant marital effects were found for height and DBP, which, respectively, explain 17% and 16% of the phenotypic variance in the HTK cohort (Tables S2–S9). The marital effect may be related to natural/positive/negative selection and, in particular, assortative mating is known to occur for height [23, 24]. In addition, we found significant common sibling effects (% total phenotypic variance) for BMI, 0.94 (10%); log TG, 0.03 (11%); TCHL, 151.28 (12%); HDL, 9.63 (7%); LDL, 141.50 (16%); and SBP, 23.27 (10%) in the HTK cohort; and HDL, 17.57 (16%) in the ASF cohort (Tables S2–S9). These significantly large percentages indicate a tendency for the environmental elements common to siblings to be similar.

However, even though ASSOC can detect the presence of some environmental effects shared by family members, the heritability estimates it produces for family-based samples are still much larger than those produced by GCTA from population-based samples. Examination of the familial correlations (Table S11) provides evidence that heritability estimated with family-based samples may be inflated if, unlike the analysis we performed with ASSOC, the sibling and marital correlations are ignored. First, the mother-father correlations for height, BMI, and DBP are significantly larger than 0 at the 0.05 significance level, whereas the usual polygenic model assumes that their correlations are 0. At the same time, in large pedigrees this positive mother-father correlation could lead to inflated parent-offspring correlations, but this effect cannot be completely handled in the existing software. Even though ASSOC can allow for a mother-father correlation, the parent-offspring correlation could be larger than expected as a result of the positive mother-father correlation; to allow completely for this, the polygenic variance should be allowed to decrease from one generation to the next. The larger correlations between siblings than those between parents and offspring could thus conceivably be partially attributable to this. Second, correlations between siblings are much larger than those between parents and offspring. In particular for log TG, TCHL, and LDL, this occurs even though the mother-father correlations are around 0. If we assume that dominant polygenic effects are small, the environmental effect shared by siblings seems to be larger than that shared by parents and offspring. The program ASSOC in S.A.G.E. appropriately allows for both a marital correlation and a common sibling component of variance over and above that due to an additive polygenic variance, though that variance is assumed to be constant across generations.

Table 3 shows correlations between DZ and MZ twins that were estimated with the linear mixed model. The correlations between MZ twins are expected to be around twice as large as those between DZ twins in the absence of both environmental effects shared by family members and dominant polygenic effects. However, for all traits other than BMI, twice the correlation between DZ twins is much larger than the correlation between MZ twins. *ρ*_*c*_ shows that the proportion of variance explained by shared environment for height may be 69.4%, and we can conclude that the correlations generated by the environmental effects shared by family members are usually much more substantial than we expect.

### 4.3. Underestimation of Heritability in Population-Based Samples

**Figure 1:**
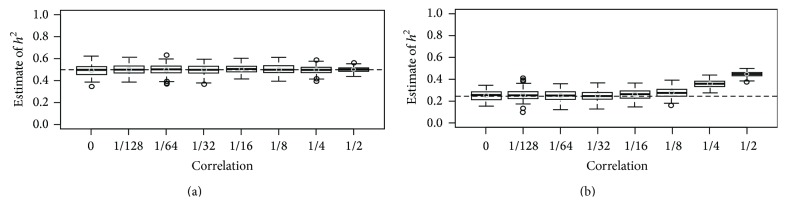
Heritability estimates for various levels of genetic correlation with 10,000 individuals when *h* ^2^ was set at 0.5 and all causal variants were generated from *U*(0,0.1). We generated 5,000 pairs of individuals with 100,000 SNPs, and each box-plot was generated with results from 200 replicates. The dashed horizontal line indicates the proportion of the total phenotypic variance explained by the SNPs used for calculating the GRM, and the estimates of heritability with GCTA are plotted against the correlation between family members. In (a), 100 causal SNPs were used to estimate the GRM, and in (b), 50 randomly selected causal SNPs were used. The horizontal dotted line indicates the relative proportion of variance explained by the SNPs.

**Figure 2:**
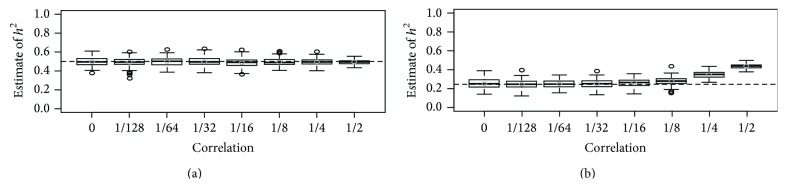
Heritability estimates for various levels of genetic correlation with 10,000 individuals when *h* ^2^ was set at 0.5 and all causal variants were generated from *U*(0.1, 0.4). We generated 5,000 pairs of individuals with 100,000 SNPs, and each box-plot was generated with results from 200 replicates. The dashed horizontal line indicates the proportion of the total phenotypic variance explained by the SNPs used for calculating the GRM, and the estimates of heritability with GCTA are plotted against the correlation between family members. In (a), 100 causal SNPs were used to estimate the GRM, and in (b), 50 randomly selected causal SNPs were used. The horizontal dotted line indicates the relative proportion of variance explained by the SNPs.

Figures 1-2 show heritability with GCTA using the GRM estimated from 100 K simulated SNPs. All causal variants were generated from *U*(0,0.1) or *U*(0.1,0.4), respectively. Each case was summarized with 200 replicates, and in Figures 1-2, we assumed that the number of causal SNPs was 100 and *h* ^2^ was set at 0.5. The results show that heritability estimates are always around the proportion of variances explained by all causal variants, 0.5, when all the causal SNPs are used to estimate the GRM. However, when half the causal SNPs are used to estimate the GRM and *r* is larger than 0.125, heritability estimates are overestimated. There is a tendency for the overestimation to be proportional to *r*. Figures 1-2 also show that the interquartile distance for a heritability estimate is inversely related to *r*. In Supplementary Figures  3–6  *h* ^2^ was assumed to be 0.1, 0.3, 0.7, or 0.9, respectively, and we found that our results are the same as in Figures 1-2.

## 5. Discussion

As a simple dimensionless overall measure of the importance of genetic factors, heritability has been used to determine the potential for predicting the genetic risk of disease. Estimating heritability requires information about genetic or familial relationships to parameterize the variance component explained by genetic factors, and formerly this was feasible only with family-based samples. With the advance of genotyping technology, large-scale genome-wide data has enabled estimation of the GRM from population-based samples, and now both family-based and population-based samples can be utilized to estimate heritability.

Heritability is a population-specific and trait-specific parameter, so it is natural that estimates have been diverse, depending on the samples and traits studied. However, we found substantial differences between the heritability estimates from population-based samples and those from family-based samples for the same trait even though both came from the same country. Although the significant differences between the two heritability estimates might be explained by heterogeneity between the samples, the estimates using population-based samples must be understood as the relative proportion of variance explained by the SNPs used to estimate the GRM [8], and this fact has been utilized to explain the missing heritability. Unbiased heritability estimation requires some individuals with large genotype correlations and the degree of genetic relationship between the individuals studied can be a more influential factor when estimating heritability. Furthermore, we attempted to quantify the variance attributable to common environment with MZ/DZ twins and found that the amount of heritability inflation can be substantial. For instance, the proportion of variance generated by shared environment is 69.4% for height, which indicates that the large value of previously reported heritability estimates for height may be generated by a large common environment component. If extended families are utilized, the amount of overestimation seems to be less substantial, but further investigation of appropriate statistical methods and study design is necessary on how to prevent the inflation of heritability estimates due to common environment effects.

Of course, in spite of our comprehensive analysis, there are several limitations to our conclusions. First, we estimated the amount of variance attributable to common environment by assuming its equivalence between MZ and DZ twins which, depending on the trait, may not be true; and there may be heteroscedasticity between MZ and DZ twins, or between twins and nontwins. If we have available MZ twins who lived apart, more accurate estimates for the variance attributable to common environment may be obtainable. Second, phenotypic differences between populations can be induced by genetic and/or environmental differences, and under population substructure the phenotypic covariance can be inflated if there are phenotypic differences between populations attributable to environmental differences. Third, it has been shown that epistasis can inflate the additive polygenic variance [1] but it is unclear whether our conclusions are still preserved in such cases. Further studies for better study design and statistical algorithms are necessary to clarify these issues.

Heritability has been a useful measure to motivate genetic studies and many statistical algorithms have been implemented to estimate it. However, complex traits result from a complex interplay of genotype and environment, and any model used to estimate heritability has a limited meaning because of the so-called phantom heritability [1]. Therefore we can conclude that it may not be always good to trust current estimates under the study designs and methodologies employed so far.

## 6. Conclusion

We estimated the heritability of traits related to cardiovascular disease, from both family-based and population-based samples, collected in Korea, and substantial differences were found between the family-based and population-based samples when using genetic markers to estimate relationship. With extensive simulations, we found that the meaning of heritability estimates can be different depending on the correlations between individuals. Furthermore, we identified the amount of variance attributable to common environment with twins and found that heritability inflation can be substantial, which indicates heritability estimates should be interpreted with caution.

## Supplementary Material

Supplementary Table S1: presents correlations between Type 2 diabetes status and various traits related to cardiovascular disease in the KARE cohort. Supplementary Tables S2-S9: contain heritability estimates for height, BMI, log(TG), TCHL, HDL, LDL, SBP and DBP. Heritability in three Korean populations was estimated with S.A.G.E. and GCTA, and variance-covariance matrices for their linear mixed models were parameterized with FRM and GRM respectively. Supplementary Table S10: presents missing rates for height, BMI, log(TG), TCHL, HDL, LDL, SBP and DBP in each cohort. Supplementary Table S11: contain correlations between family members. The mother-father, parent-offspring and sibling correlations, and their 95% confidence intervals were estimated. Supplementary Figures S1-S2: present Q-Q plots for height, BMI, log(TG), TCHL, HDL, LDL, SBP and DBP in each cohort. Supplementary Figures S3-S6: present heritability estimates for various levels of genetic correlation with 10,000 individuals when heritability was assumed to be 0.1, 0.3, 0.7, or 0.9.

## Figures and Tables

**Table 1:** Descriptive statistics for the traits in each cohort.

Trait	HTK (*n* = 1801)			ASF (*n* = 784)			KARE (*n* = 8842)		
	1st Q	Median	3rd Q	1st Q	Median	3rd Q	1st Q	Median	3rd Q
Sex (m/f)	711/1090 (0.39/0.61)			372/412 (0.47/0.53)			4183/4659 (0.47/0.653)		
Age	35	43	57	35	46.5	60	44	50	60
Height	155.6	160.9	167.7	155.4	162.1	169	153.3	159.7	166.6
BMI	21.47	23.61	25.9	22.21	24.39	26.64	22.51	24.48	26.5
log⁡TG	4.17	4.55	4.94	4.36	4.71	5.106	4.605	4.913	5.252
HDL	41	48	57	37	43	51	37	44	50
LDL	91	110	132	93.6	115	135.3	114.2	115.7	136.4
SBP	108	118.7	130	110	120	130	104.67	115.33	128
DBP	70	72	80	72	79	84	68.67	74	81.33
TCHL	164	187	211	165	185	210.2	167	189	214

**Table 2:** Estimates (s.e.) of heritability.

		Cohort			
		Family-based		Population-based	All
		HTK	ASF	KARE
Traits	Height	0.76 (0.04)	0.66 (0.09)	0.32 (0.04)	0.60 (0.02)
	BMI	0.43 (0.05)	0.41 (0.08)	0.15 (0.04)	0.32 (0.02)
	TG	0.37 (0.05)	0.27 (0.08)	0.21 (0.04)	0.24 (0.02)
	TCHL	0.47 (0.05)	0.50 (0.08)	0.18 (0.04)	0.30 (0.02)
	HDL	0.72 (0.04)	0.50 (0.07)	0.16 (0.04)	0.38 (0.02)
	LDL	0.43 (0.05)	0.47 (0.08)	0.16 (0.04)	0.29 (0.02)
	SBP	0.37 (0.05)	0.23 (0.08)	0.26 (0.04)	0.23 (0.02)
	DBP	0.53 (0.05)	0.21 (0.08)	0.21 (0.04)	0.24 (0.02)

**Table 3:** Estimates of variance components in the HTK cohort. MZ and DZ twins were separated out and used to estimate correlations of MZ and DZ twins. *ρ*_*c*_ indicates a lower bound for the proportion of variance explained by the environmental effects shared by family members.

	cor (MZ)^a^	cor (DZ)^b^	*ρ*_*c* (95% confidence interval)_
Height	0.970	0.832	0.694 (0.690, 0.698)
BMI	0.729	0.232	0.266 (0.061, 0.496)
log⁡TG	0.551	0.336	0.121 (0.070, 0.172)
TCHL	0.624	0.382	0.139 (0.093, 0.185)
HDL	0.677	0.476	0.275 (0.240, 0.310)
LDL	0.656	0.342	0.028 (−0.022, 0.078)
SBP	0.601	0.453	0.306 (0.268, 0.344)
DBP	0.646	0.585	0.524 (0.501, 0.547)

^a^Correlation of MZ twins. ^b^Correlation of DZ twins.
