# Supplementary material for: On the Estimation of Heritability with Family-Based and Population-Based Samples
Source: Biomed Res Int. 2015 Aug 3;2015:671349. doi: 10.1155/2015/671349 (PMC4538414; doi:10.1155/2015/671349)
Supplement: Supplementary file 1 — Supplementary Table S1: presents correlations between Type 2 diabetes status and various traits related to cardiovascular disease in the KARE cohort. Supplementary Tables S2-S9: contain heritability estimates for height, BMI, log(TG), TCHL, HDL, LDL, SBP and DBP. Heritability in three Korean populations was estimated with S.A.G.E. and GCTA, and variance-covariance matrices for their linear mixed models were parameterized with FRM and GRM respectively. Supplementary Table S10: presents missing rates for height, BMI, log(TG), TCHL, HDL, LDL, SBP and DBP in each cohort. Supplementary Table S11: contain correlations between family members. The mother-father, parent-offspring and sibling correlations, and their 95% confidence intervals were estimated. Supplementary Figures S1-S2: present Q-Q plots for height, BMI, log(TG), TCHL, HDL, LDL, SBP and DBP in each cohort. Supplementary Figures S3-S6: present heritability estimates for various levels of genetic correlation with 10,000 individuals when heritability was assumed to be 0.1, 0.3, 0.7, or 0.9. [file 671349.f1.pdf]

SUPPLEMENTARY MATERIALS

Table S1 Correlation between Type 2 diabetes status and various traits related to cardiovascular disease in the KARE cohort.

Trait	correlation
**Height**	0.017
**BMI**	0.096
**Log TG**	0.154
**TCHL**	0.114
**HDL**	-0.047
**LDL**	0.070
**SBP**	0.112
**DBP**	0.076

Table S2 Estimates of heritability for height. Heritability of height in three Korean populations were estimated. S.A.G.E. estimates the heritability with FRM and GCTA does it with GRM. The additive polygenic variance, random error variance and phenotypic variance are denoted by σ_a^2, σ^2 and σ_p^2, respectively. Contrary to GCTA, S.A.G.E. can also estimate the relative proportion of variance explained by common sibling effects and common marital effects, and those are denoted by σ_m^2 and σ_s^2, respectively. The standard errors of each estimate are shown in parenthesis. If the variance components are not estimable, the results are denoted as NA.

**Cohort**	**Family**						**Population**		**ALL**	
	**HTK**			**ASF**			**KARE**			
**Program**	**GCTA w/o cutoff**	**GCTA w/ cutoff**	**S.A.G.E.**	**GCTA w/o cutoff**	**GCTA w/ cutoff**	**S.A.G.E.**	**GCTA w/o cutoff**	**GCTA w/ cutoff**	**GCTA w/o cutoff**	**GCTA w/ cutoff**
h^2**(s.e.)**	0.76(0.04)	0.78(0.46)	0.81(0.06)	0.66(0.07)	0.79(0.64)	0.67(0.09)	0.36(0.03)	0.32(0.04)	0.60(0.02)	0.32(0.04)
σ_a^2**(s.e.)**	21.81(1.71)	22.30(13.38)	23.31(2.21)	19.86(2.87)	23.60(21.49)	19.14(3.12)	10.29(1.01)	8.98(1.22)	17.34(0.75)	9.12(1.06)
σ^2 **(s.e.)**	6.86(1.04)	6.32(13.15)	0.50(2.87)	10.34(1.90)	6.33(18.69)	3.84(3.55)	18.03(0.96)	19.37(1.19)	11.51(0.57)	19.24(1.03)
σ_m^2**(s.e.)**	NA	NA	4.83(2.05)	NA	NA	3.05(2.25)	NA	NA	NA	NA
σ_s^2**(s.e.)**	NA	NA	0.28(0.91)	NA	NA	2.49(1.54)	NA	NA	NA	NA
σ_p^2 **(s.e.)**	28.67(1.10)	28.62(1.53)	28.92(1.15)	30.20(1.75)	29.93(3.57)	28.51(1.63)	28.32(0.43)	28.35(0.48)	28.85(0.41)	28.36(0.45)
n	1,791	717	1,799	784	342	784	8,842	7,178	11,421	8,172

Table S3 Estimates of heritability for BMI. Heritability of BMI in three Korean populations were estimated. S.A.G.E. estimates the heritability with FRM and GCTA does it with GRM. The additive polygenic variance, random error variance and phenotypic variance are denoted by σ_a^2, σ^2 and σ_p^2, respectively. Contrary to GCTA, S.A.G.E. can also estimate the relative proportion of variance explained by common sibling effects and common marital effects, and those are denoted by σ_m^2 and σ_s^2, respectively. The standard errors of each estimate are shown in parenthesis. If the variance components are not estimable, the results are denoted as NA.

Cohort	Family						Population		ALL	
	HTK			ASF			KARE			
**Program**	**GCTA w/o cutoff**	**GCTA w/ cutoff**	**S.A.G.E.**	**GCTA w/o cutoff**	**GCTA w/ cutoff**	**S.A.G.E.**	**GCTA w/o cutoff**	**GCTA w/ cutoff**	**GCTA w/o cutoff**	**GCTA w/ cutoff**
h^2(s.e.)	0.43(0.05)	0(0.43)	0.38(0.06)	0.41(0.08)	0.95(0.65)	0.43(0.09)	0.18(0.03)	0.15(0.04)	0.32(0.02)	0.14(0.04)
σ_a^2(s.e.)	4.31(0.55)	0(4.31)	3.73(0.68)	4.53(1.04)	10.84(8.57)	4.53(1.03)	1.71(0.32)	1.43(0.4)	3.14(0.23)	1.32(0.34)
σ^2 (s.e.)	5.77(0.46)	9.96(4.33)	4.70(0.91)	6.55(0.83)	0.63(7.43)	5.22(1.52)	7.84(0.33)	8.21(0.41)	6.71(0.22)	8.27(0.36)
σ_m^2(s.e.)	NA	NA	0.43(0.71)	NA	NA	0.73(1.04)	NA	NA	NA	NA
σ_s^2(s.e.)	NA	NA	0.94(0.42)	NA	NA	NA	NA	NA	NA	NA
σ_p^2 (s.e.)	10.08(0.36)	9.96(0.53)	9.81(0.36)	11.08(0.61)	11.47(1.41)	10.48(0.56)	9.56(0.14)	9.63(0.16)	9.86(0.13)	9.58(0.15)
n	1,789	717	1,789	784	342	784	8,838	7,177	11,415	8,171

Table S4 Estimates of heritability for log(TG). Heritability of log(TG) in three Korean populations were estimated. S.A.G.E. estimates the heritability with FRM and GCTA does it with GRM. The additive polygenic variance, random error variance and phenotypic variance are denoted by σ_a^2, σ^2 and σ_p^2, respectively. Contrary to GCTA, S.A.G.E. can also estimate the relative proportion of variance explained by common sibling effects and common marital effects, and those are denoted by σ_m^2 and σ_s^2, respectively. The standard errors of each estimate are shown in parenthesis. If the variance components are not estimable, the results are denoted as NA.

**Cohort**	**Family**						**Population**		**ALL**	
	**HTK**			**ASF**			**KARE**			
**Program**	**GCTA w/o cutoff**	**GCTA w/ cutoff**	**S.A.G.E.**	**GCTA w/o cutoff**	**GCTA w/ cutoff**	**S.A.G.E.**	**GCTA w/o cutoff**	**GCTA w/ cutoff**	**GCTA w/o cutoff**	**GCTA w/ cutoff**
h^2**(s.e.)**	0.37(0.05)	0.16(0.42)	0.29(0.07)	0.27(0.08)	0.39(0.66)	0.27(0.10)	0.19(0.03)	0.21(0.04)	0.24(0.02)	0.19(0.04)
σ_a^2**(s.e.)**	0.10(0.02)	0.05(0.13)	0.08(0.02)	0.07(0.02)	0.10(0.18)	0.07(0.03)	0.05(0.01)	0.05(0.01)	0.06(0.01)	0.05(0.01)
σ^2 **(s.e.)**	0.17(0.01)	0.25(0.13)	0.17(0.01)	0.20(0.02)	0.15(0.15)	0.15(0.04)	0.19(0.01)	0.19(0.01)	0.19(0.01)	0.2(0.01)
σ_m^2**(s.e.)**	NA	NA	NA	NA	NA	0.02(0.03)	NA	NA	NA	NA
σ_s^2**(s.e.)**	NA	NA	0.03(0.01)	NA	NA	0.02(0.02)	NA	NA	NA	NA
σ_p^2 **(s.e.)**	0.27(0.01)	0.3(0.02)	0.27(0.01)	0.27(0.01)	0.25(0.03)	0.26(0.01)	0.24(0)	0.24(0)	0.24(0.00)	0.24(0.00)
n	1,769	710	1,765	776	339	776	8,841	7,177	11,386	8,164

Table S5 Estimates of heritability for TCHL. Heritability of TCHL in three Korean populations were estimated. S.A.G.E. estimates the heritability with FRM and GCTA does it with GRM. The additive polygenic variance, random error variance and phenotypic variance are denoted by σ_a^2, σ^2 and σ_p^2, respectively. Contrary to GCTA, S.A.G.E. can also estimate the relative proportion of variance explained by common sibling effects and common marital effects, and those are denoted by σ_m^2 and σ_s^2, respectively. The standard errors of each estimate are shown in parenthesis. If the variance components are not estimable, the results are denoted as NA.

**Cohort**	**Family**						**Population**		**ALL**	
	**HTK**			**ASF**			**KARE**			
**Program**	**GCTA w/o cutoff**	**GCTA w/ cutoff**	**S.A.G.E.**	**GCTA w/o cutoff**	**GCTA w/ cutoff**	**S.A.G.E.**	**GCTA w/o cutoff**	**GCTA w/ cutoff**	**GCTA w/o cutoff**	**GCTA w/ cutoff**
h^2**(s.e.)**	0.47(0.05)	0.58(0.44)	0.42(0.06)	0.50(0.08)	1.00(0.64)	0.48(0.10)	0.24(0.03)	0.18(0.04)	0.30(0.02)	0.14(0.04)
σ_a^2**(s.e.)**	591.91(68.92)	790.29(607.99)	516.78(86.65)	641.03(126.02)	1477.97(1083.31)	594.55 (136.58)	303.82(43.32)	226.86(53.05)	380.53(29.70)	184.49(45.92)
σ^2 **(s.e.)**	667.42(54.90)	571.5(600.76)	460.95(101.97)	645.15(94.74)	0.00(939.87)	515.27(107.30)	986.61(43.57)	1067.44(54.19)	901.60(28.39)	1115.85(47.84)
σ_m^2**(s.e.)**	NA	NA	104.29(70.80)	NA	NA	NA	NA	NA	NA	NA
σ_s^2**(s.e.)**	NA	NA	151.28(49.75)	NA	NA	123.25(80.79)	NA	NA	NA	NA
σ_p^2 **(s.e.)**	1259.33(44.95)	1361.8(72.37)	1233.30(45.60)	1286.18(72.90)	1477.98(179.48)	1233.06(68.31)	1290.43(19.53)	1294.30(21.68)	1282.14(17.33)	1300.34(20.39)
n	1,797	718	1,793	784	342	784	8,841	7,177	11,422	8,172

Table S6 Estimates of heritability for HDL. Heritability of HDL in three Korean populations were estimated. S.A.G.E. estimates the heritability with FRM and GCTA does it with GRM. The additive polygenic variance, random error variance and phenotypic variance are denoted by σ_a^2, σ^2 and σ_p^2, respectively. Contrary to GCTA, S.A.G.E. can also estimate the relative proportion of variance explained by common sibling effects and common marital effects, and those are denoted by σ_m^2 and σ_s^2, respectively. The standard errors of each estimate are shown in parenthesis. If the variance components are not estimable, the results are denoted as NA.

**Cohort**	**Family**						**Population**		**ALL**	
	**HTK**			**ASF**			**KARE**			
**Program**	**GCTA w/o cutoff**	**GCTA w/ cutoff**	**S.A.G.E.**	**GCTA w/o cutoff**	**GCTA w/ cutoff**	**S.A.G.E.**	**GCTA w/o cutoff**	**GCTA w/ cutoff**	**GCTA w/o cutoff**	**GCTA w/ cutoff**
h^2**(s.e.)**	0.72(0.04)	0(0.44)	0.74(0.06)	0.50(0.07)	0.74(0.63)	0.52(0.09)	0.18(0.03)	0.16(0.04)	0.38(0.02)	0.15(0.04)
σ_a^2**(s.e.)**	105.26(8.6)	0(66.16)	110.21(11.51)	63.73(11.24)	106.88(101.72)	58.86(11.73)	18.15(3.41)	16.5(4.17)	42.01(2.59)	15.17(3.77)
σ^2 **(s.e.)**	41.31(5.45)	151.13(66.27)	14.99(14.28)	62.48(8.46)	38.17(88.58)	35.32(14.10)	82.63(3.5)	83.63(4.27)	68.27(2.34)	89.28(3.91)
σ_m^2**(s.e.)**	NA	NA	13.14(10.03)	NA	NA	1. 24(9.39)	NA	NA	NA	NA
σ_s^2**(s.e.)**	NA	NA	9.63(5.04)	NA	NA	17.57(7.30)	NA	NA	NA	NA
σ_p^2 **(s.e.)**	146.57(5.54)	151.13(8.02)	147.98(5.89)	126.21(7.03)	145.06(17.03)	112.99(6.27)	100.78(1.52)	100.14(1.68)	110.28(1.51)	104.45(1.64)
n	1,791	715	1,787	784	342	784	8,841	7,177	11,416	8,169

Table S7 Estimates of heritability for LDL. Heritability of LDL in three Korean populations were estimated. S.A.G.E. estimates the heritability with FRM and GCTA does it with GRM. The additive polygenic variance, random error variance and phenotypic variance are denoted by σ_a^2, σ^2 and σ_p^2, respectively. Contrary to GCTA, S.A.G.E. can also estimate the relative proportion of variance explained by common sibling effects and common marital effects, and those are denoted by σ_m^2 and σ_s^2, respectively. The standard errors of each estimate are shown in parenthesis. If the variance components are not estimable, the results are denoted as NA.

**Cohort**	**Family**						**Population**		**ALL**	
	**HTK**			**ASF**			**KARE**			
**Program**	**GCTA w/o cutoff**	**GCTA w/ cutoff**	**S.A.G.E.**	**GCTA w/o cutoff**	**GCTA w/ cutoff**	**S.A.G.E.**	**GCTA w/o cutoff**	**GCTA w/ cutoff**	**GCTA w/o cutoff**	**GCTA w/ cutoff**
h^2**(s.e.)**	0.43(0.05)	1.00(0.45)	0.33(0.06)	0.47(0.08)	0.54(0.67)	0.52(0.11)	0.22(0.03)	0.16(0.04)	0.29(0.02)	0.15(0.04)
σ_a^2**(s.e.)**	377.20(48.08)	932.96(426.29)	291.25(62.08)	488.50(101.10)	639.35(849.86)	518.66(128.85)	225.08(35.58)	168.85(43.55)	292.71(24.13)	151.57(37.55)
σ^2 **(s.e.)**	508.98(39.87)	0(416.46)	436.39(75.66)	549.83(77.66)	539.27(743.94)	309.48(147.15)	812.75(36.06)	866.91(44.61)	725.57(23.19)	877.79(38.97)
σ_m^2**(s.e.)**	NA	NA	17.70(55.51)	NA	NA	103.76(85.81)	NA	NA	NA	NA
σ_s^2**(s.e.)**	NA	NA	141.50(39.31)	NA	NA	66.17(66.35)	NA	NA	NA	NA
σ_p^2 **(s.e.)**	886.18(31.40)	932.96(49.98)	886.84(32.43)	1038.33(58.45)	1178.61(139.22)	998.07(56.25)	1037.84(15.93)	1035.76(17.56)	1018.28(13.91)	1029.35(16.33)
n	1,791	715	1,787	784	342	784	8,578	6,998	11,156	7,989

Table S8 Estimates of heritability for SBP. Heritability of SBP in three Korean populations were estimated. S.A.G.E. estimates the heritability with FRM and GCTA does it with GRM. The additive polygenic variance, random error variance and phenotypic variance are denoted by σ_a^2, σ^2 and σ_p^2, respectively. Contrary to GCTA, S.A.G.E. can also estimate the relative proportion of variance explained by common sibling effects and common marital effects, and those are denoted by σ_m^2 and σ_s^2, respectively. The standard errors of each estimate are shown in parenthesis. If the variance components are not estimable, the results are denoted as NA.

**Cohort**	**Family**						**Population**		**ALL**	
	**HTK**			**ASF**			**KARE**			
**Program**	**GCTA w/o cutoff**	**GCTA w/ cutoff**	**S.A.G.E.**	**GCTA w/o cutoff**	**GCTA w/ cutoff**	**S.A.G.E.**	**GCTA w/o cutoff**	**GCTA w/ cutoff**	**GCTA w/o cutoff**	**GCTA w/ cutoff**
h^2**(s.e.)**	0.37(0.05)	0.89(0.46)	0.35(0.06)	0.23(0.08)	0(0.76)	0.22(0.08)	0.22(0.03)	0.26(0.04)	0.23(0.02)	0.20(0.04)
σ_a^2**(s.e.)**	87.87(12.76)	228.84(120.42)	79.82(15.84)	45.21(16.38)	0(155.97)	42.00(14.98)	62.03(9.43)	74.54(11.81)	61.71(6.10)	57.85(10)
σ^2 **(s.e.)**	148.45(11.09)	28.46(118)	124.22(19.50)	151.14(15.33)	205.73(138)	147.29(15.20)	222.16(9.56)	208.42(11.72)	212.42(6.16)	224.36(10.19)
σ_m^2**(s.e.)**	NA	NA	2.47(13.91)	NA	NA	NA	NA	NA	NA	NA
σ_s^2**(s.e.)**	NA	NA	23.27(9.90)	NA	NA	NA	NA	NA	NA	NA
σ_p^2 **(s.e.)**	236.32(8.29)	257.3(13.75)	229.78(8.31)	196.35(10.36)	205.73(24.44)	189.29(9.69)	284.19(4.30)	282.96(4.76)	274.13(3.67)	282.21(4.43)
n	1,791	717	1,787	784	342	784	8,841	7,177	11,416	8,171

Table S9 Estimates of heritability for DBP. Heritability of DBP in three Korean populations were estimated. S.A.G.E. estimates the heritability with FRM and GCTA does it with GRM. The additive polygenic variance, random error variance and phenotypic variance are denoted by σ_a^2, σ^2 and σ_p^2, respectively. Contrary to GCTA, S.A.G.E. can also estimate the relative proportion of variance explained by common sibling effects and common marital effects, and those are denoted by σ_m^2 and σ_s^2, respectively. The standard errors of each estimate are shown in parenthesis. If the variance components are not estimable, the results are denoted as NA.

**Cohort**	**Family**						**Population**		**ALL**	
	**HTK**			**ASF**			**KARE**			
**Program**	**GCTA w/o cutoff**	**GCTA w/ cutoff**	**S.A.G.E.**	**GCTA w/o cutoff**	**GCTA w/ cutoff**	**S.A.G.E.**	**GCTA w/o cutoff**	**GCTA w/ cutoff**	**GCTA w/o cutoff**	**GCTA w/ cutoff**
h^2**(s.e.)**	0.53(0.05)	0.12(0.44)	0.60(0.06)	0.21(0.08)	0.24(0.67)	0.18(0.09)	0.21(0.03)	0.21(0.04)	0.24(0.02)	0.14(0.03)
σ_a^2**(s.e.)**	55.52(5.97)	12.51(44.62)	62.54(7.77)	16.78(6.76)	19.34(56.18)	13.90(7.13)	25.61(4.21)	26.99(5.41)	28.78(2.75)	17.78(4.45)
σ^2 **(s.e.)**	50.1(4.52)	89.96(44.69)	21.39(9.28)	63.10(6.39)	61.88(49.6)	59.24(10.17)	98.78(4.28)	101.66(5.45)	91.12(2.73)	108.50(4.64)
σ_m^2**(s.e.)**	NA	NA	16.95(6.85)	NA	NA	0.96(6.70)	NA	NA	NA	NA
σ_s^2**(s.e.)**	NA	NA	3.72(3.66)	NA	NA	3.97(5.96)	NA	NA	NA	NA
σ_p^2 **(s.e.)**	105.61(3.83)	102.47(5.43)	104.60(3.94)	79.88(4.21)	81.23(9.23)	78.07(4.01)	124.38(1.88)	128.65(2.16)	119.90(1.61)	126.28(1.98)
n	1,792	717	1,788	784	342	784	8,841	7,177	11,417	8,171

Table S10 Missing rates for each phenotype

	Cohort		
	**KARE**	**HTK**	**ASF**
**Height**	0%	0.11%	0%
**BMI**	0.05%	0.22%	0%
**LogTG**	0.01%	1.56%	1.02%
**TCHL**	0.01%	0%	0%
**HDL**	0.01%	0.33%	0%
**LDL**	2.95%	0.33%	0%
**SBP**	0.01%	0.33%	0%
**DBP**	0.01%	0.28%	0%

Table S11 Empirical correlations between family members. The mother-father, parent-offspring and sibling correlations, and their 95% confidence intervals were estimated with FCOR in S.A.G.E.

Cohorts	Types	Height	BMI	LogTG	TCHL	HDL	LDL	SBP	DBP
**HTK**	**Mother – Father**	0.172 ±0.142	0.212 ±0.136	-0.085 ±0.149	0.084 ±0.147	0.106 ±0.143	0.069 ±0.152	0.046 ±0.153	0.227 ±0.152
	**Parent – offspring**	0.413 ±0.063	0.197 ±0.068	0.114 ±0.062	0.169 ±0.068	0.363 ±0.064	0.164 ±0.069	0.175 ±0.074	0.297 ±0.075
	**Sibling**	0.517 ±0.056	0.331 ±0.591	0.290 ±0.060	0.367 ±0.060	0.480 ±0.058	0.382 ±0.060	0.283 ±0.057	0.374 ±0.060
**ASF**	**Mother – Father**	0.144 ±0.181	0.075 ±0.222	0.033 ±0.209	-0.091 ±0.218	0.052 ±0.187	-0.007 ±0.226	0.134 ±0.179	0.039 ±0.184
	**Parent – offspring**	0.298 ±0.102	0.215 ±0.090	0.118 ±0.099	0.219 ±0.090	0.237 ±0.100	0.190 ±0.091	0.161 ±0.116	0.140 ±0.108
	**Sibling**	0.353 ±0.128	0.168 ±0.123	0.245 ±0.175	0.264 ±0.129	0.350 ±0.130	0.236 ±0.127	0.177 ±0.156	0.080 ±0.115

Figure S1 Q-Q plots for HEIGHT, BMI, LOGTG and HDL in each cohort.

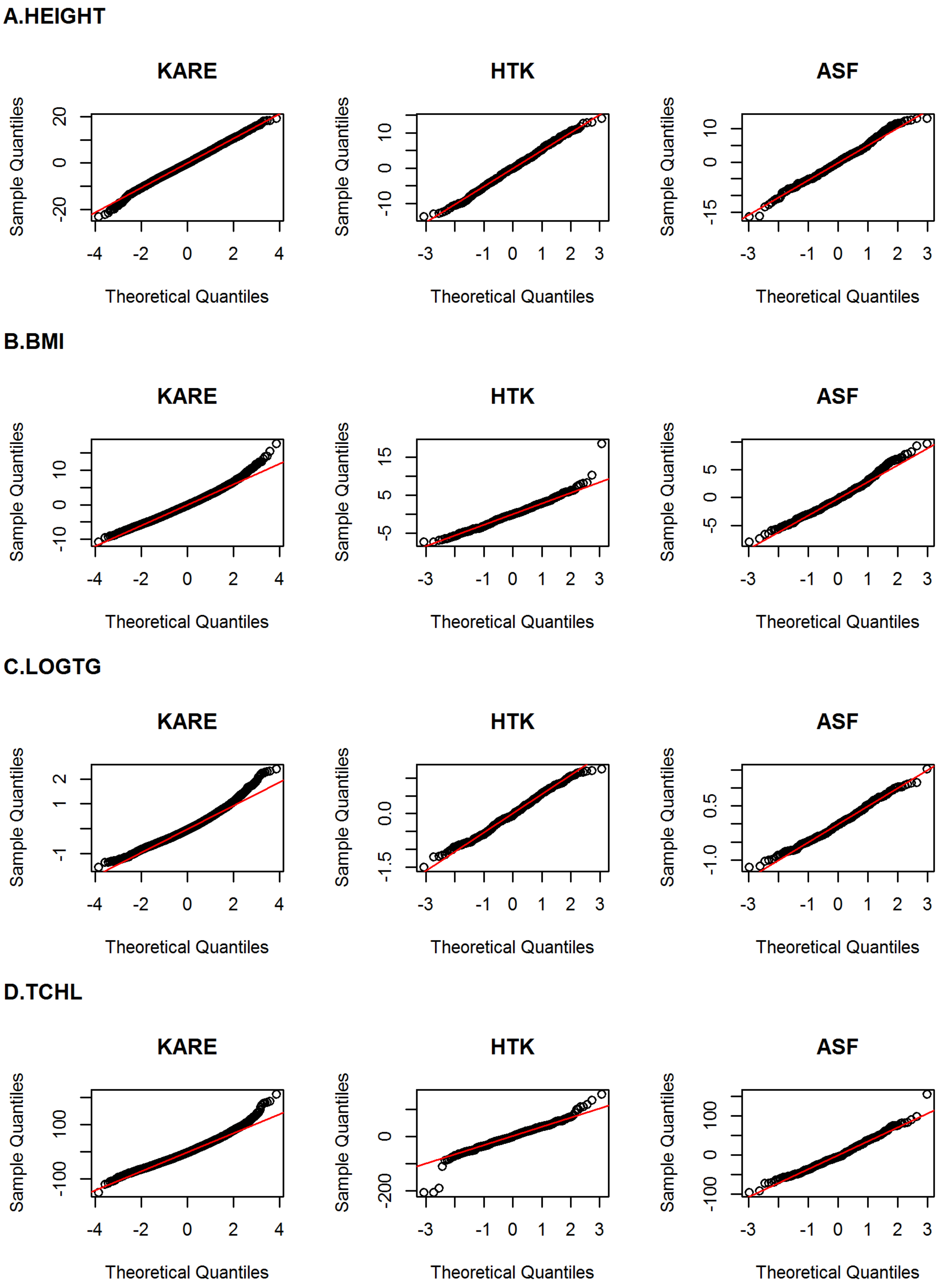

Figure S2 Q-Q plots for LDL, SBP, DBP and TCHL in each cohort.

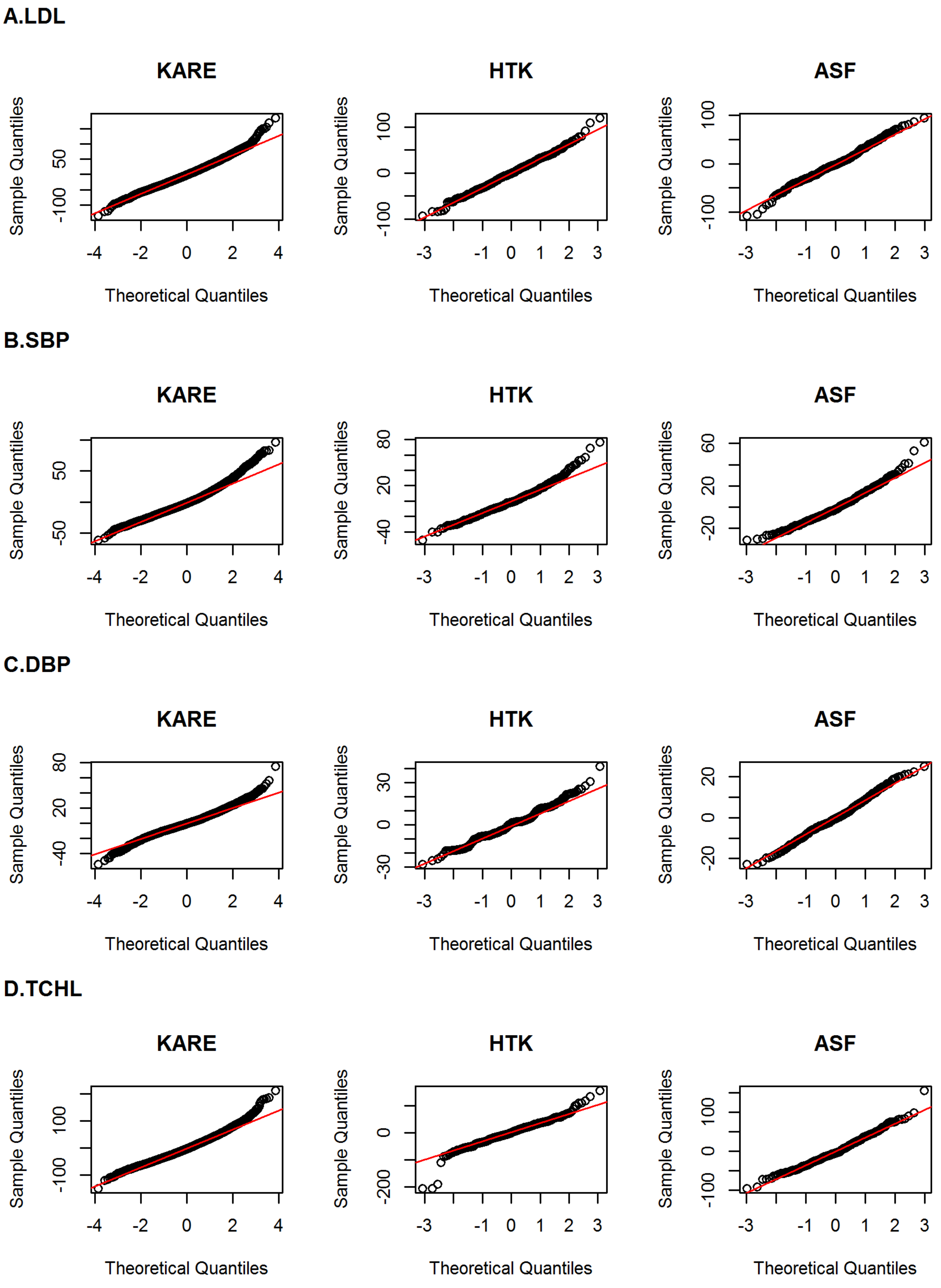

Figure S3 Heritability estimates for various levels of genetic correlation with 10,000 individuals when h^2 was set at 0.1. We generated 5,000 pairs of individuals with 100,000 SNPs, and each box-plot was generated with results from 200 replicates. The dashed horizontal line indicates the proportion of the total phenotypic variance explained by the SNPs used for calculating the GRM, and the estimates of heritability with GCTA are plotted against the correlation between family members. In **a**, all causal variants were generated from U(0, 0.1) and 100 causal SNPs were used to estimate the GRM, in **b**, all causal variants were generated from U(0, 0.1) and 50 causal SNPs were used, in **c**, all causal variants were generated from U(0.1, 0.4) and 100 causal SNPs were used, and in **d**, all causal variants were generated from U(0.1, 0.4) and 50 causal SNPs were used. The horizontal dotted line indicates the relative proportion of variance explained by the SNPs.

a

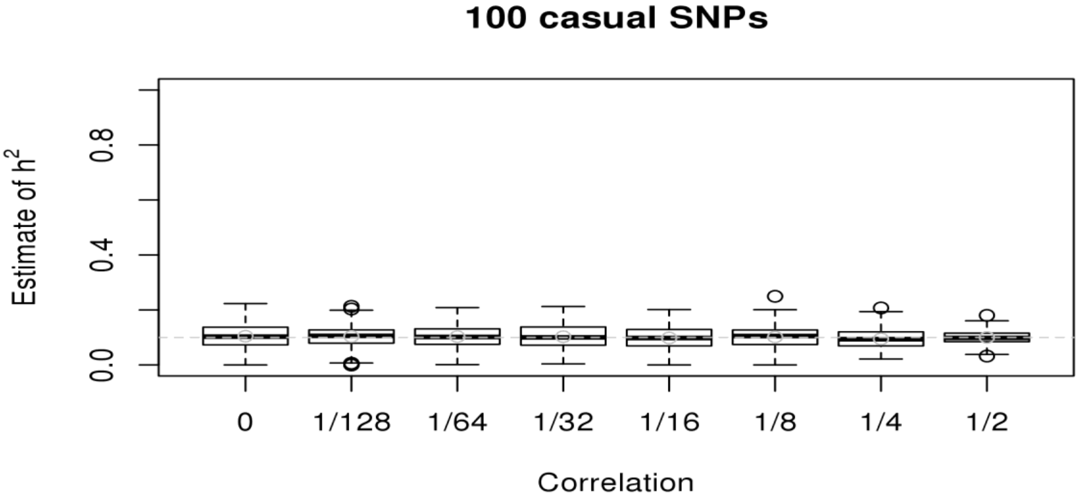

b

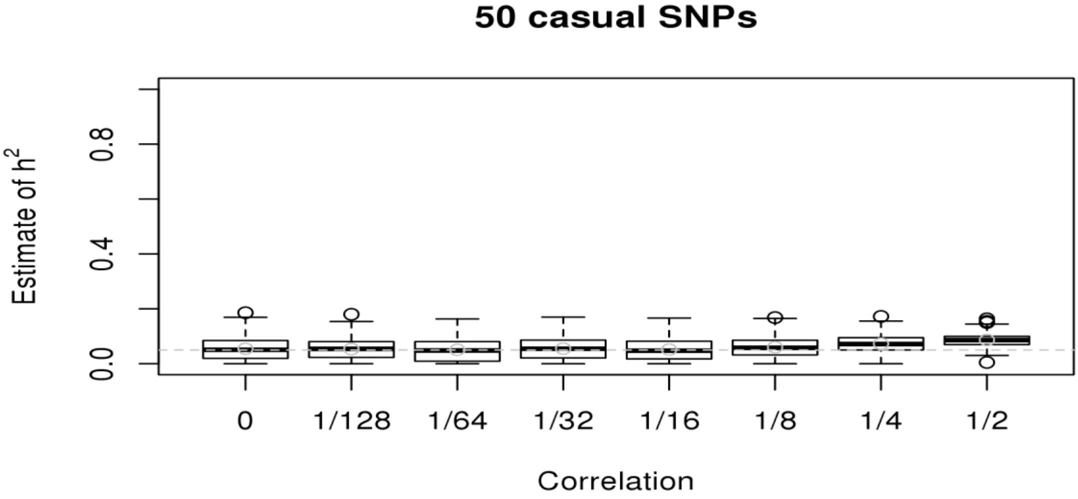

c

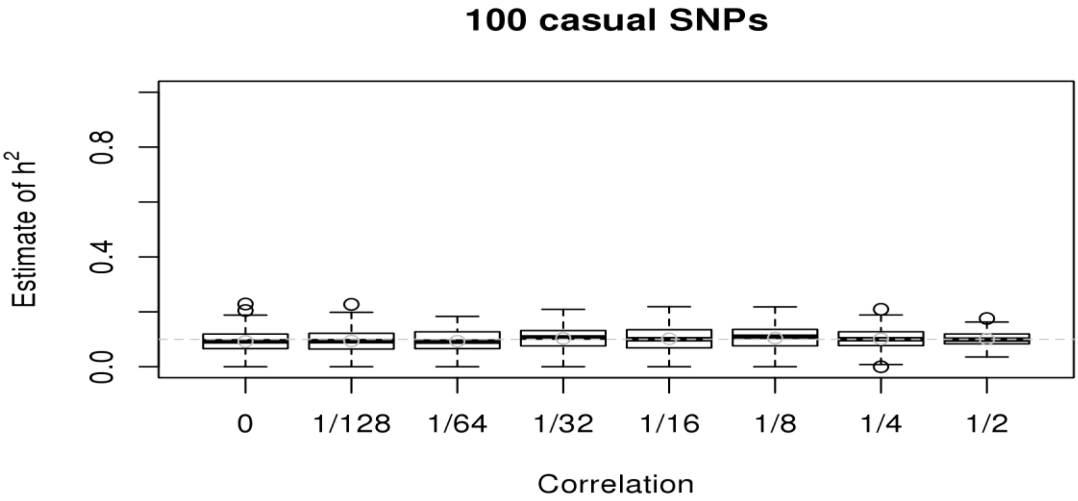
100 casual SNPs
Estimate of h2
0.0
0.4
0.8
0
1/128
1/64
1/32
1/16
1/8
1/4
1/2
Correlation

d

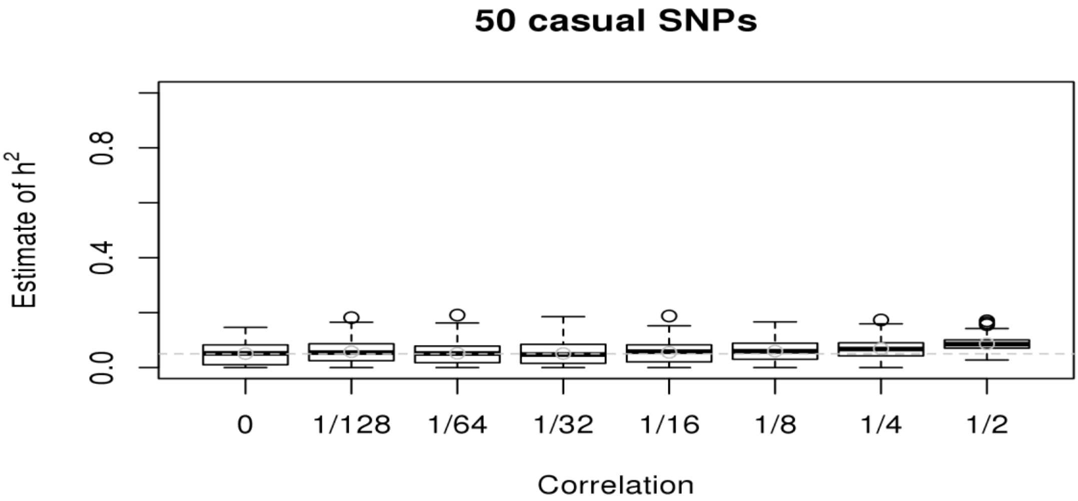
50 casual SNPs
Estimate of h2
0.0
0.4
0.8
0
1/128
1/64
1/32
1/16
1/8
1/4
1/2
Correlation

Figure S4 Heritability estimates for various levels of genetic correlation with 10,000 individuals when h^2 was set at 0.3. We generated 5,000 pairs of individuals with 100,000 SNPs, and each box-plot was generated with results from 200 replicates. The dashed horizontal line indicates the proportion of the total phenotypic variance explained by the SNPs used for calculating the GRM, and the estimates of heritability with GCTA are plotted against the correlation between family members. In **a**, all causal variants were generated from U(0, 0.1) and 100 causal SNPs were used to estimate the GRM, in **b**, all causal variants were generated from U(0, 0.1) and 50 causal SNPs were used, in **c**, all causal variants were generated from U(0.1, 0.4) and 100 causal SNPs were used, and in **d**, all causal variants were generated from U(0.1, 0.4) and 50 causal SNPs were used. The horizontal dotted line indicates the relative proportion of variance explained by the SNPs.

a

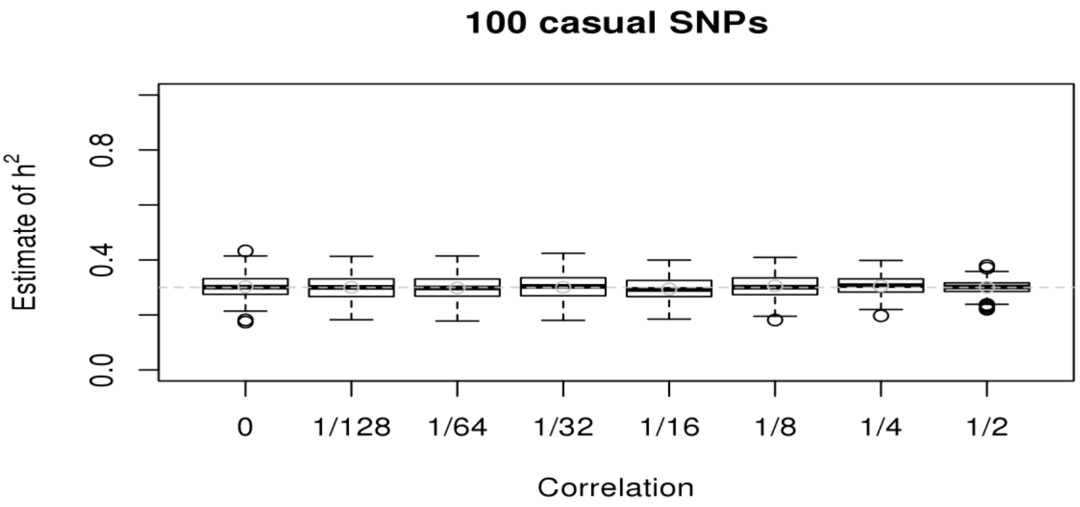

b

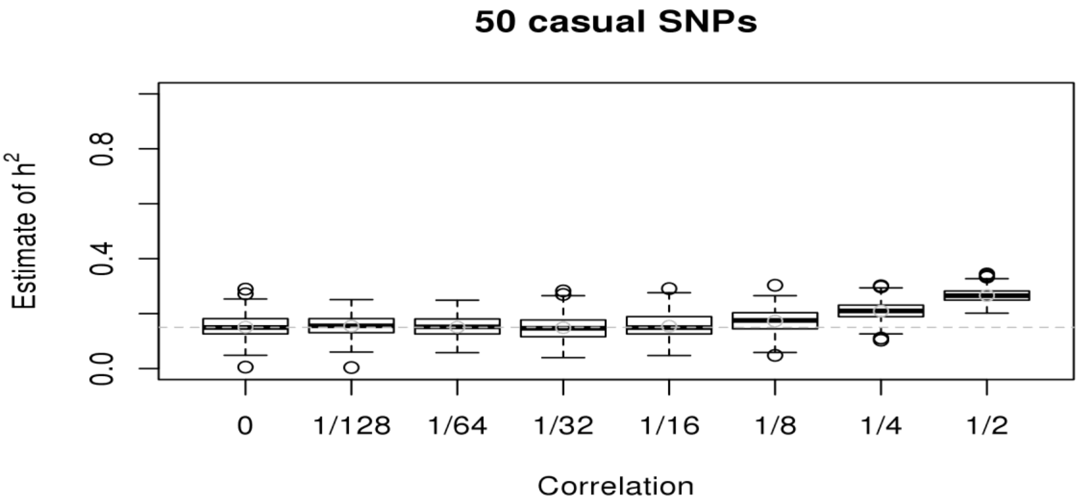

c

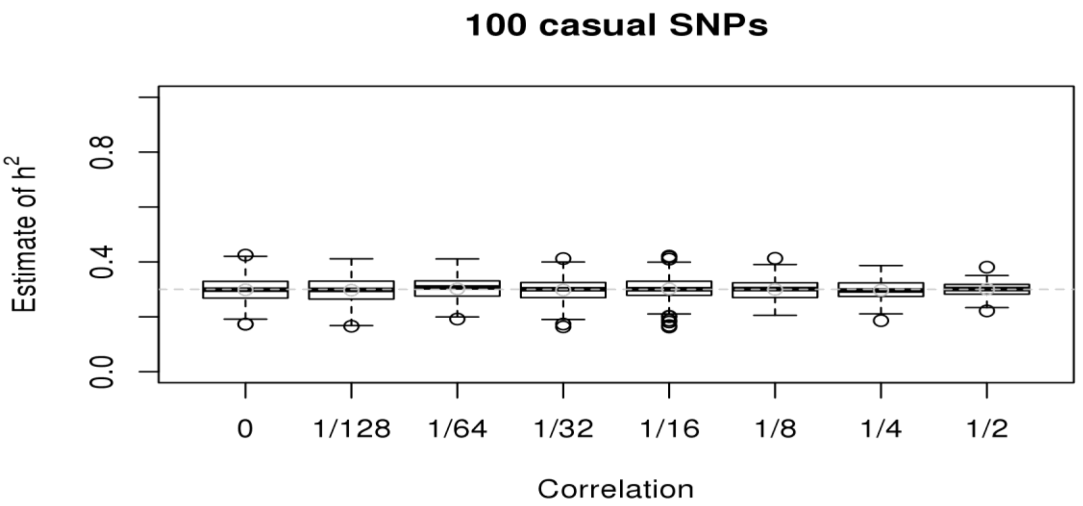
100 casual SNPs
Estimate of h2
0.0
0.4
0.8
0
1/128
1/64
1/32
1/16
1/8
1/4
1/2
Correlation

d

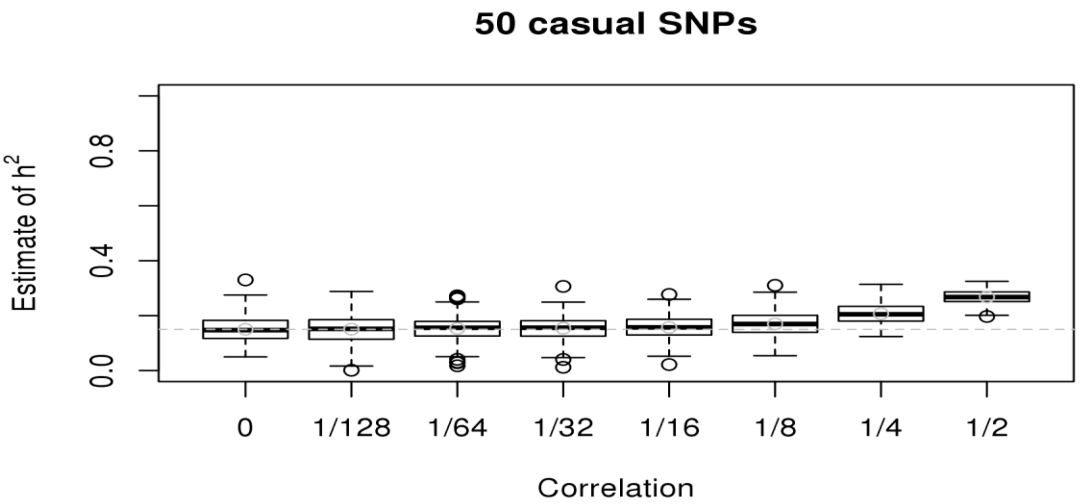
50 casual SNPs
Estimate of h2
0.0
0.4
0.8
0
1/128
1/64
1/32
1/16
1/8
1/4
1/2
Correlation

Figure S5 Heritability estimates for various levels of genetic correlation with 10,000 individuals when h^2 was set at 0.7. We generated 5,000 pairs of individuals with 100,000 SNPs, and each box-plot was generated with results from 200 replicates. The dashed horizontal line indicates the proportion of the total phenotypic variance explained by the SNPs used for calculating the GRM, and the estimates of heritability with GCTA are plotted against the correlation between family members. In **a**, all causal variants were generated from U(0, 0.1) and 100 causal SNPs were used to estimate the GRM, in **b**, all causal variants were generated from U(0, 0.1) and 50 causal SNPs were used, in **c**, all causal variants were generated from U(0.1, 0.4) and 100 causal SNPs were used, and in **d**, all causal variants were generated from U(0.1, 0.4) and 50 causal SNPs were used. The horizontal dotted line indicates the relative proportion of variance explained by the SNPs.

a

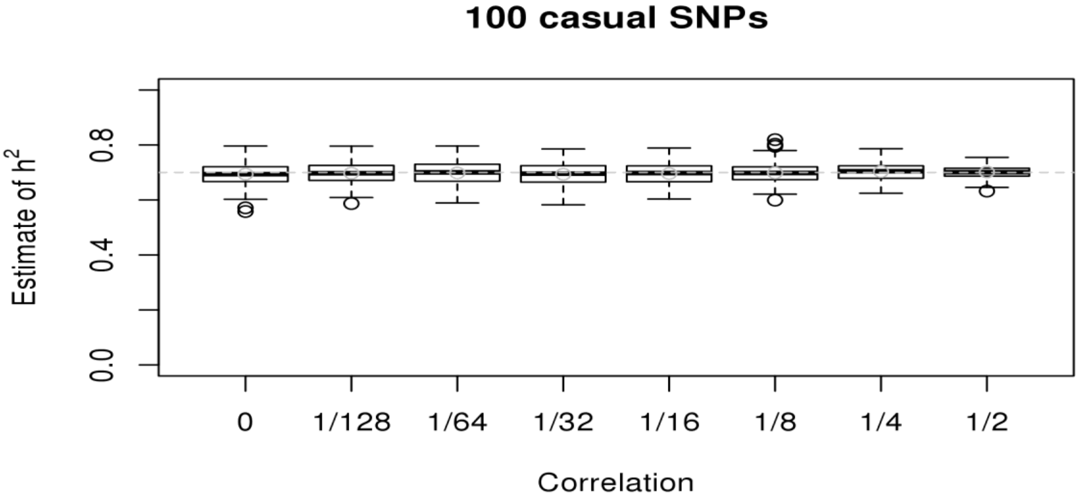

b

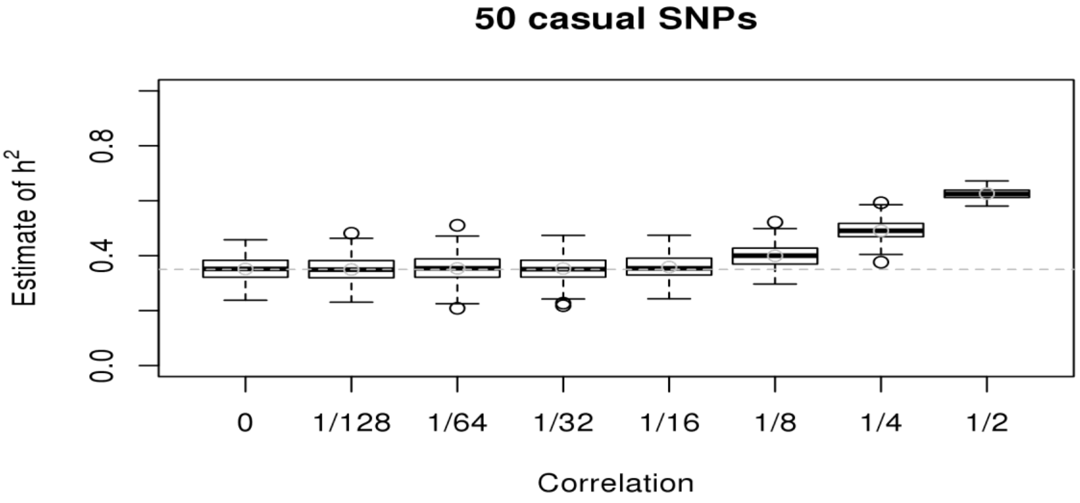

c

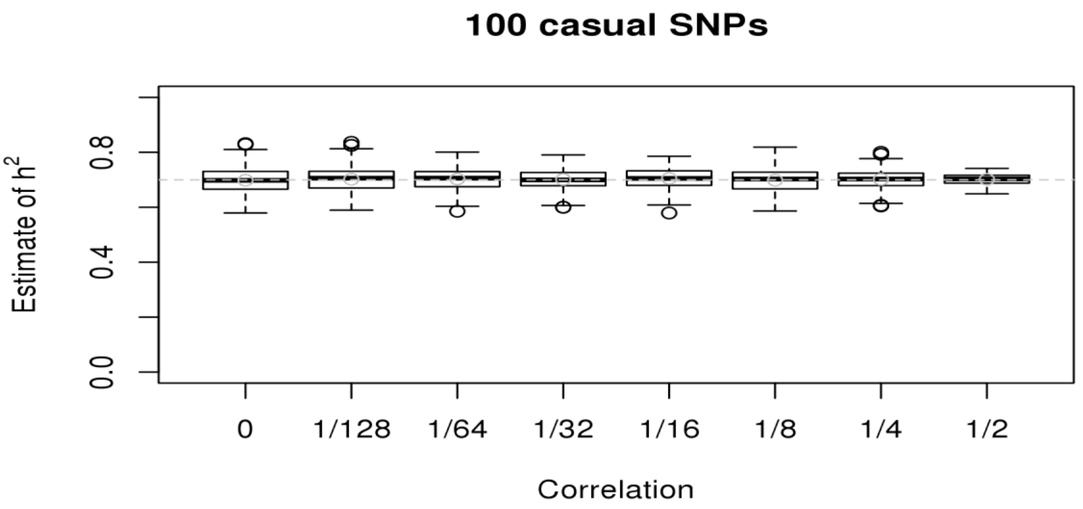
100 casual SNPs
Estimate of h2
0.0
0.4
0.8
0
1/128
1/64
1/32
1/16
1/8
1/4
1/2
Correlation

d

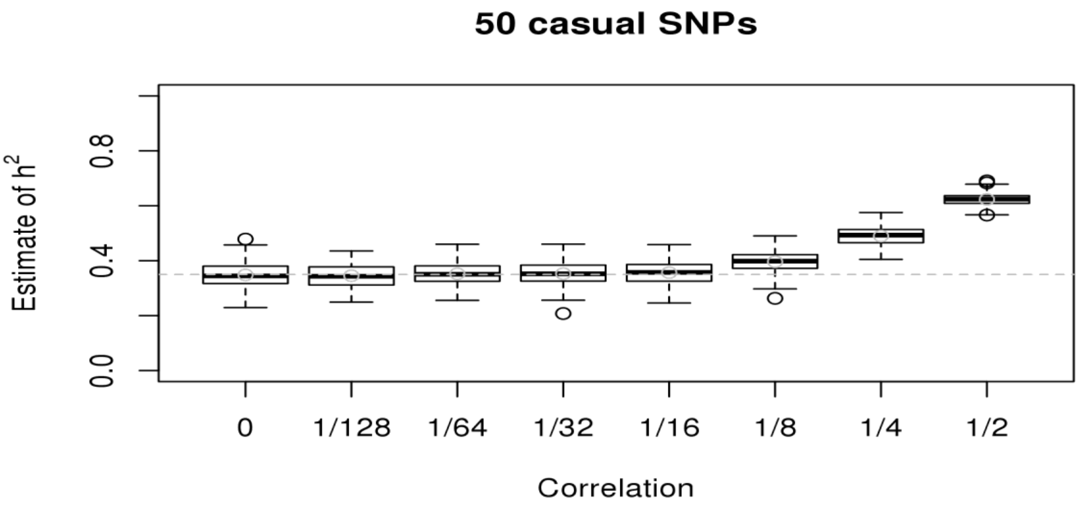
50 casual SNPs
Estimate of h2
0.0
0.4
0.8
0
1/128
1/64
1/32
1/16
1/8
1/4
1/2
Correlation

Figure S6 Heritability estimates for various levels of genetic correlation with 10,000 individuals when h^2 was set at 0.9. We generated 5,000 pairs of individuals with 100,000 SNPs, and each box-plot was generated with results from 200 replicates. The dashed horizontal line indicates the proportion of the total phenotypic variance explained by the SNPs used for calculating the GRM, and the estimates of heritability with GCTA are plotted against the correlation between family members. In **a**, all causal variants were generated from U(0, 0.1) and 100 causal SNPs were used to estimate the GRM, in **b**, all causal variants were generated from U(0, 0.1) and 50 causal SNPs were used, in **c**, all causal variants were generated from U(0.1, 0.4) and 100 causal SNPs were used, and in **d**, all causal variants were generated from U(0.1, 0.4) and 50 causal SNPs were used. The horizontal dotted line indicates the relative proportion of variance explained by the SNPs.

a

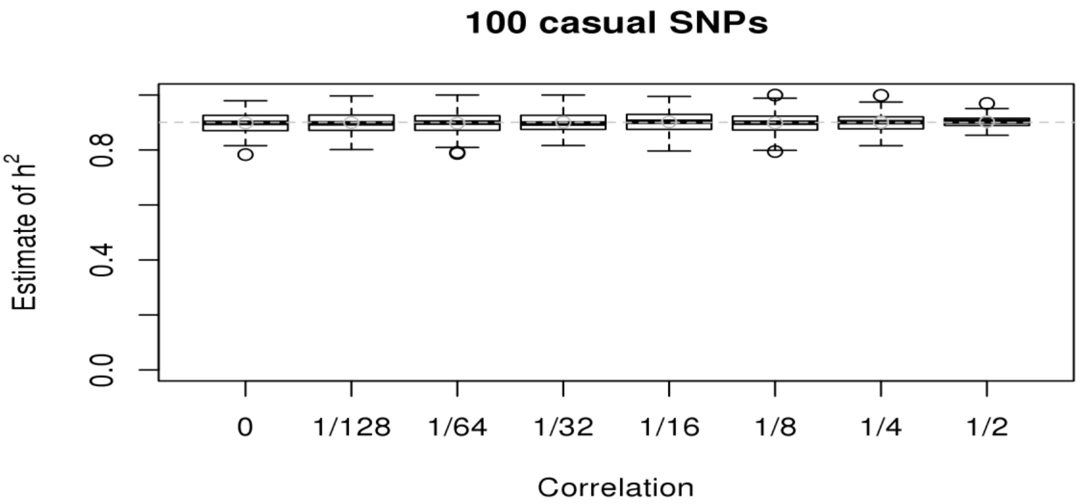

b

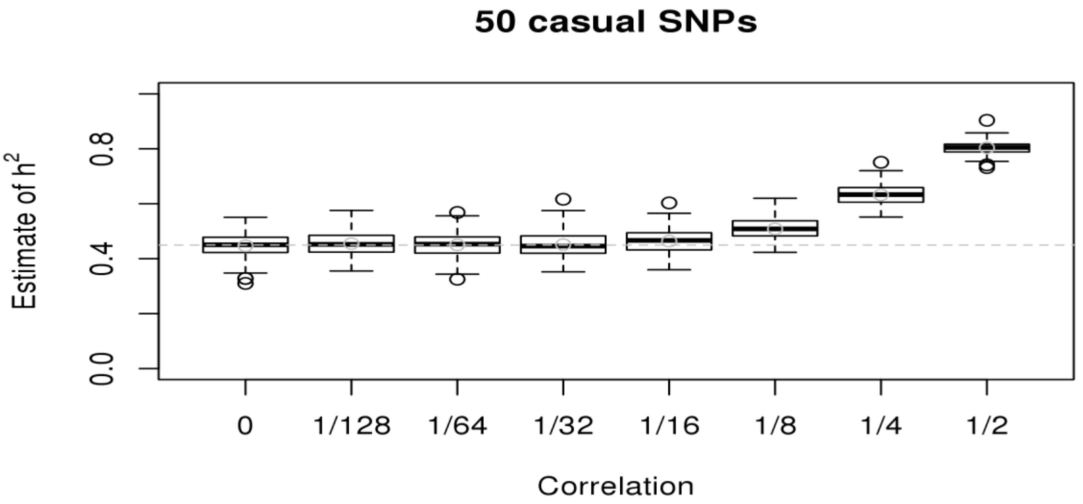

c

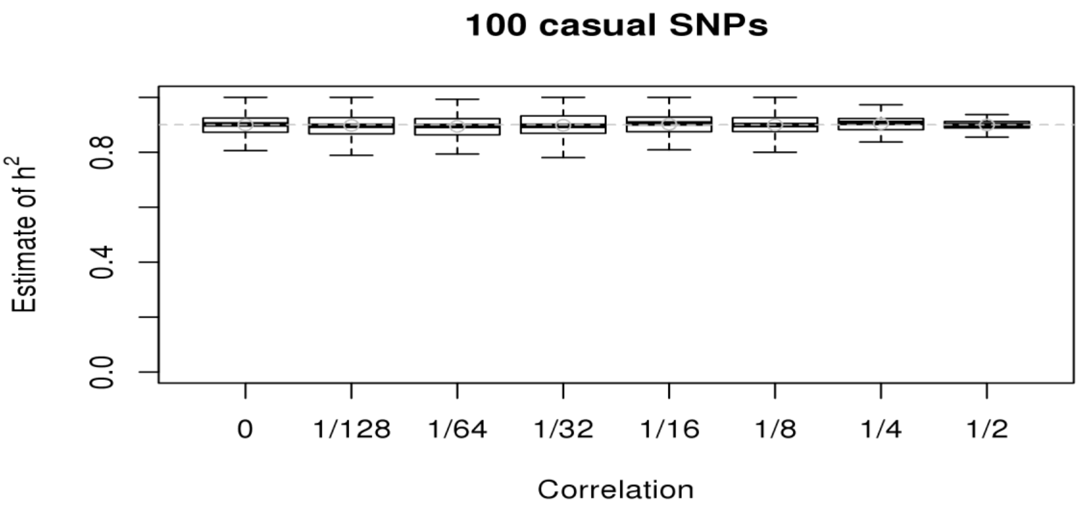
100 casual SNPs
Estimate of h2
0.0
0.4
0.8
0
1/128
1/64
1/32
1/16
1/8
1/4
1/2
Correlation

d

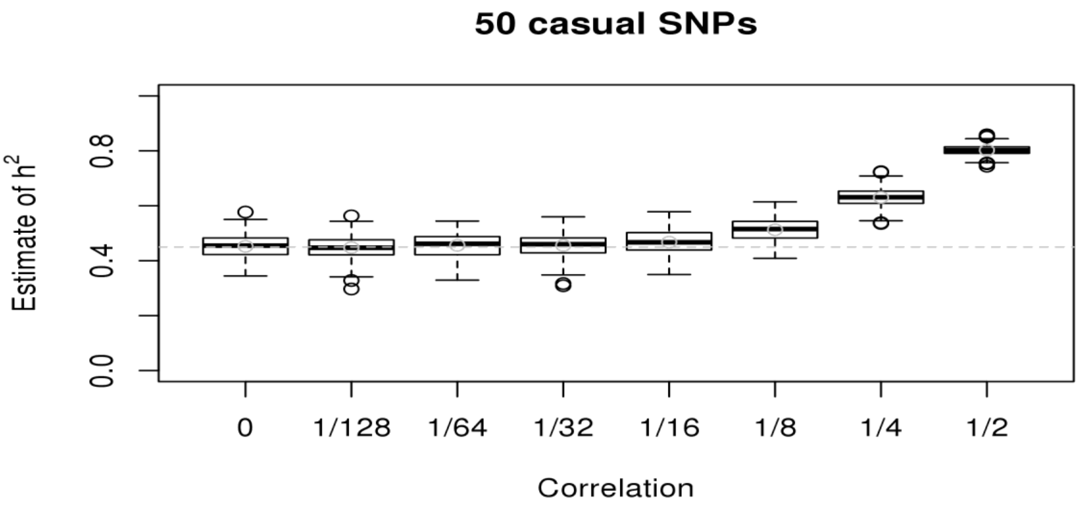
50 casual SNPs
Estimate of h2
0.0
0.4
0.8
0
1/128
1/64
1/32
1/16
1/8
1/4
1/2
Correlation
